# Reply: Factors Influencing Central Nervous System Abnormalities in m.11778G>A Carriers

**DOI:** 10.3390/brainsci10080518

**Published:** 2020-08-05

**Authors:** Cezary Grochowski, Kamil Jonak

**Affiliations:** 1 Laboratory of Virtual Man, Medical University of Lublin, 20-400 Lublin, Poland; 2 Department of Biomedical Engineering, Lublin University of Technology, 20-618 Lublin, Poland; jonak.kamil@gmail.com; 3 Department of Psychiatry, Psychotherapy and Early Intervention, Medical University of Lublin, 20-439 Lublin, Poland

First of all, I would like to thank the reader for their interest and taking the time to analyze our work [1]. We are very pleased that this paper induced academic discussion. Thank you for all the comments, which I am sure will improve the quality of the paper and clarify several aspects of the paper. I would like to specify and discuss several doubts made by the reader’s letter [2].

In the course of the whole study we have scanned 21 patients with LHON (Leber’s hereditary optic neuropathy) disease. Six of those individuals were receiving idebenone at the time of the study and three were qualified for the therapy yet were not receiving the drug. We decided to exclude those individuals to analyze a more homogenous group. Because of the fact that a relatively small group of patients were receiving Raxone therapy, we did not perform the statistical analysis on this group. 

To the best of our knowledge, none of the patients reported receiving any additional treatment of the ocular disease or using specific dietary restrictions, including a ketogenic diet. In fact, there are several papers reporting the advantages the ketogenic diet performed on cell lines or animals, including the paper cited by Prof. Finsterer, however those papers usually included a small number of analyzed specimens.

Sadly, not all of the patients were able to provide full clinical details regarding their disease. It has to be underlined that, often, it was the first MRI scan they had in their life so such an analysis was impossible to perform. Among the analyzed group five of the patients were single family members and the remaining ten individuals did not have any family history of LHON disease. It has to be underlined that during statistical analysis we used data obtained from age-matched, healthy controls. Moreover, this exploratory methodological investigation to the best of our knowledge is the first application of submillimeter high-field MRI for the evaluation of morphometric changes in LHON patients. Obviously, the study has several limitations, which were listed at the end of the study (Paragraph 4.3). Further study should be done in this important and interesting patient group, including as many clinical details as possible.
